# What distinguishes adolescents with suicidal thoughts from those who have attempted suicide? A population‐based birth cohort study

**DOI:** 10.1111/jcpp.12878

**Published:** 2018-03-01

**Authors:** Becky Mars, Jon Heron, E. David Klonsky, Paul Moran, Rory C. O'Connor, Kate Tilling, Paul Wilkinson, David Gunnell

**Affiliations:** ^1^ Population Health Sciences University of Bristol Bristol UK; ^2^ University of British Columbia Vancouver BC Canada; ^3^ NIHR Bristol Biomedical Research Centre University Hospitals Bristol NHS Foundation Trust and University of Bristol Bristol UK; ^4^ Suicidal Behaviour Research Laboratory Institute of Health & Wellbeing University of Glasgow Glasgow UK; ^5^ Department of Psychiatry University of Cambridge Cambridge UK; ^6^ Cambridgeshire and Peterborough NHS Foundation Trust Cambridge UK

**Keywords:** Suicide attempt, ideation, suicidal thoughts, ALSPAC, self‐harm

## Abstract

**Background:**

Only one‐third of young people who experience suicidal ideation attempt suicide. It is important to identify factors which differentiate those who attempt suicide from those who experience suicidal ideation but do not act on these thoughts.

**Methods:**

Participants were 4,772 members of the Avon Longitudinal Study of Parents and Children (ALSPAC), a UK population‐based birth cohort. Suicide ideation and attempts were assessed at age 16 years via self‐report questionnaire. Multinomial regression was used to examine associations between factors that differentiated adolescents in three groups: no suicidal ideation or attempts, suicidal ideation only and suicide attempts. Analyses were conducted on an imputed data set based on those with complete outcome data (suicidal thoughts and attempts) at age 16 years (*N* = 4,772).

**Results:**

The lifetime prevalence of suicidal ideation and attempts in the sample was 9.6% and 6.8% respectively. Compared to adolescents who had experienced suicidal ideation, those who attempted suicide were more likely to report exposure to self‐harm in others (adjusted OR for family member self‐harm: 1.95, for friend self‐harm: 2.61 and for both family and friend self‐harm: 5.26). They were also more likely to have a psychiatric disorder (adjusted OR for depression: 3.63; adjusted OR for anxiety disorder: 2.20; adjusted OR for behavioural disorder: 2.90). Other risk factors included female gender, lower IQ, higher impulsivity, higher intensity seeking, lower conscientiousness, a greater number of life events, body dissatisfaction, hopelessness, smoking and illicit drug use (excluding cannabis).

**Conclusions:**

The extent of exposure to self‐harm in others and the presence of psychiatric disorder most clearly differentiate adolescents who attempt suicide from those who only experience suicidal ideation. Further longitudinal research is needed to explore whether these risk factors predict progression from suicidal ideation to attempts over time.

## Abstract 2

Read the Commentary on this article at doi: 10.1111/jcpp.12982

## Introduction

Thoughts of suicide (suicidal ideation) are common in adolescents and are a well‐established risk factor for suicidal behaviour (Nock et al., 2008). Although many risk and protective factors for suicidal behaviour have been identified, little is known about the factors that differentiate those most likely to attempt suicide from those who only think about suicide. Recent findings from epidemiological and meta‐analytical studies suggest that many well‐established risk factors for suicide (including depression, hopelessness and impulsivity) strongly predict the development of suicidal ideation, but only weakly predict attempts among those thinking about suicide (Kessler, Borges, & Walters, 1999; May & Klonsky, 2016; Nock et al., 2009, 2013). Identifying factors that best distinguish between these groups of adolescents has important implications for both clinical practice and suicide theory.

Recent theoretical models of suicide, including the interpersonal theory (IPT) (Van Orden et al., 2010), the integrated motivational–volitional (IMV) model (O'Connor, 2011) and the three‐step theory (3ST) (Klonsky & May, 2015) all fit within an ‘ideation to action’ framework (Klonsky, Qiu, & Saffer, 2017), which posits that the development of suicidal ideation and progression from ideation to attempts are distinct processes with separate risk factors and explanations. According to the IPT, perceptions of low belongingness and high burdensomeness contribute to desire for suicide, but acquired capability for suicide is required to facilitate a potentially lethal attempt. The IMV model proposes that defeat and entrapment (termed ‘motivational factors’) increase the likelihood that suicidal ideation will emerge, and that a collection of ‘volitional’ factors (e.g. acquired capability, access to lethal means, exposure to suicidal behaviour, impulsivity) explains the propensity to act on suicidal thoughts. The 3ST is the most recently developed theory and suggests that suicidal ideation and attempts are explained by a combination of four factors: pain, hopelessness, connectedness and suicide capability (dispositional, learned and practical).

Suicide capability (i.e. the degree to which an individual feels able to make a suicide attempt) is a key determinant in each of the three models. Factors thought to be related to this concept include increased fearlessness about death; persistence through pain and distress; exposure to self‐harm in others; knowledge about and access to lethal means; and previous non‐suicidal self‐harm. A recent review of the literature found factors related to increased suicide capability were consistently associated with suicide attempts amongst those with ideation (Klonsky et al., 2017).

There is increasing evidence from both clinical and population‐based samples that one of these risk factors, exposure to self‐harm in others, reliably distinguishes between young people with suicidal ideation and attempts (Asarnow et al., 2008; Dhingra, Boduszek, & O'Connor, 2015; O'Connor & Nock, 2014). Other factors that have been found to be associated with suicide attempts amongst ideators in this age group include mental health problems, aggression, impulsivity, substance abuse, female gender, stressful life events, abuse, relationship problems, pregnancy events and factors relating to service use/treatment (Asarnow et al., 2008; Borges, Benjet, Medina‐Mora, Orozco, & Nock, 2008; Goldston et al., 2016; Nock et al., 2013; O'Connor, Rasmussen, & Hawton, 2012; Taliaferro & Muehlenkamp, 2014). However, effect sizes are often small, and findings have frequently been inconsistent across studies or have not been replicated. Therefore, more research is needed to understand this important issue, including the consideration of additional risk factors which have not previously been investigated using an ‘ideation to action’ approach.

Much of the existing research in this area has been limited to small and/or unrepresentative samples. Previous studies have also typically relied on retrospective reporting of the occurrence and timing of risk factors which may be subject to recall bias. The present study uses data from a large UK population‐based birth cohort to (a) explore risk factors for adolescent suicidal ideation and attempts and (b) identify factors that distinguish between these groups. We compared a wide range of recognised risk factors between three groups: those who had attempted suicide, those who had thought about suicide but never made an attempt and those without any suicide history. Our analyses were exploratory, given the limited research in this area. However, as suicide capability is a key component of the theoretical models of suicide, we hypothesised that putative risk factors related to increased suicide capability (such as exposure to self‐harm in others) would most clearly differentiate between those with a history of ideation and those who had attempted suicide.

## Methods

### Sample

The Avon Longitudinal Study of Parents and Children (ALSPAC) is a population‐based birth cohort study examining influences on health and development across the life course. The ALSPAC core enrolled sample consists of 14,541 pregnant women resident in the former county of Avon in South West England (United Kingdom), with expected delivery dates between 1 April 1991 and 31 December 1992 (Boyd et al., 2013). Of the 14,062 live births, 13,798 were singletons/firstborn of twins and were alive at 1 year of age. Participants have been followed up regularly since recruitment through questionnaires and research clinics. Information about ALSPAC is available at (http://www.bristol.ac.uk/alspac), which includes details of all available data via a fully searchable data dictionary (http://www.bris.ac.uk/alspac/researchers/data-access/data-dictionary). Ethical approval for the study was obtained from the ALSPAC Law and Ethics committee. Written informed consent was obtained after the procedure(s) had been fully explained.

The present investigation is based on the subsample of 4,772 children who completed a self‐report questionnaire on suicidal ideation and behaviours at mean age of 16 years 8 months (Kidger, Heron, Lewis, Evans, & Gunnell, 2012). The postal questionnaire was sent to 9,370 participants of whom 4,850 (51.8%) returned it, and 4,772 responded to the questions on self‐harm and suicidal ideation. Questionnaire responders were more likely than non‐responders to be female, to be white, have a lower parity, have a mother with higher education, have a higher household income and a higher parental social class; they were also less likely to have experienced overcrowding (Table S1).

### Measures

#### Outcome measure: Lifetime history of suicidal ideation and attempts

The questions included in the ALSPAC questionnaire were based on those used in the Child and Adolescent Self‐harm in Europe (CASE) study (Madge et al., 2008). Suicidal ideation was assessed with the question ‘Have you ever thought of killing yourself, even if you would not really do it?’ Response options for this question were ‘yes’ or ‘no’. Self‐harm was assessed with the question ‘Have you ever hurt yourself on purpose in any way (e.g. by taking an overdose of pills, or by cutting yourself)?’ Those who responded positively were then asked a series of follow‐up questions to establish suicidal intent. Participants were classified as having self‐harmed with suicidal intent if (a) when asked about the reasons why they hurt themselves on the most recent occasion, they selected the response option ‘I wanted to die’ or (b) they responded positively to the question ‘On any of the occasions when you have hurt yourself on purpose, have you ever seriously wanted to kill yourself?’. Individuals may self‐harm on multiple occasions for different reasons. Throughout the paper, we refer to those with a lifetime history of suicidal self‐harm as having ‘attempted suicide’, but recognise that many individuals in this group will have also engaged in episodes of non‐suicidal self‐harm.

#### Risk factors

A description of the risk factors examined in this study is provided in Table 1. The selection of risk factors was informed by psychological models of suicide and by previous literature. These risk factors are all known to be associated with self‐harm/suicide; however, many have not been examined within an ‘ideation to action’ framework before.

**Table 1:** Table of measures

Risk factors	Age of assessment	Measure used	Rater	Additional information
Demographic variables				
Female gender	Birth	Questionnaire item	Mother	
Psychosocial variables				
Total IQ	8 years	Wechsler intelligence test for children (WISC‐III) (Wechsler, Golombok, & Rust, 1992)	Child	
Executive function				
Updating	8 years	WISC‐III	Child	Digit span task
Attentional switching		The adapted Test of Everyday Attention for Children (TEA‐Ch) (Robertson, Ward, Ridgeway, & Nimmo‐Smith, 1996)	Child	The dual‐attention task of the ‘Sky‐Search’ subtest
Attentional control		The TEA‐Ch	Child	The inhibition aspect of the ‘Opposite Worlds’ task
Impulsivity	10 years	Stop‐signal task (Logan, Cowan, & Davis, 1984)	Child	
Sensation seeking	16 years	Arnett inventory of sensation‐seeking scale (Arnett, 1994)	Child	Novelty and intensity subscales
Big‐5 personality dimensions	14 years	International personality item pool (Goldberg, 1999)	Child	Five subscales (extraversion, agreeableness, conscientiousness, emotional stability and intellect/openness to experience)
Self‐harm in friends and family				
Parent suicide attempt	Repeated eight times from birth to 11 years	Questionnaire item	Mother	
Family self‐harm	16 years	Questionnaire item	Child	Lifetime rating
Friend self‐harm	16 years	Questionnaire item	Child	Lifetime rating
Extent of exposure	16 years	Created from responses to the questions on friend self‐harm and family self‐harm	Child	A three‐category variable was created for those who responded to the items on friend self‐harm and family self‐harm. Participants either (a) reported no exposure to self‐harm, (b) reported self‐harm in either a friend or a family member but not both or (c) reported self‐harm in both a friend and a family member
				
Number of life events	16 years	Life events questionnaire	Child	Since age 12
Childhood sexual abuse	Repeated seven times from birth to 8 years	Questionnaire item	Mother	
Cruelty to children in household	Repeated eight times from birth to 11 years	Questionnaire item	Mother	
Being bullied	12 years	Modified version of the bullying and friendship interview schedule (Woods & Wolke, 2003)	Child	Overt or relational bullying at least once a week over the previous 6 months
Body dissatisfaction	13 years	Questionnaire item		Unhappy or happy over the past year
Psychiatric/mental health variables				
Psychiatric disorder	15 years	DAWBA (Goodman, Ford, Richards, Gatward, & Meltzer, 2000)	Child	Depressive disorder, anxiety disorder and behavioural disorder (oppositional defiant disorder/conduct disorder/attention deficit hyperactivity disorder)
Depressive symptoms	12 years	Short mood and feelings questionnaire (Messer et al., 1995)	Child	
Hopelessness	16 years	Community Assessment of Psychic Experience (CAPE) (Konings, Bak, Hanssen, van Os, & Krabbendam, 2006)	Child	Two items used
Substance use				
Alcohol	15 years	Questionnaire items	Child	Consuming ≥4 drinks on a typical occasion in the last 6 months
Cannabis				At least occasional use
Smoking				Regular smoking (at least weekly)
Other Illicit drug use				Past year. Coded positive if the young person endorsed any of the following types of drug use: sniffing poppers, solvents, aerosols, gas or glue, using amphetamines, barbiturates, ecstasy, crack, cocaine, LSD, heroin, ketamine, spanglers, ritalin or benzodiazepines.

ALSPAC is a longitudinal study and participants have completed regular assessments since birth. The timing of risk factors was dependent on the age at which the data were collected and ranges from birth to age 16 years. Several risk factors were collected at the same time as the outcome measures (age 16 years). These included sensation seeking; exposure to self‐harm in friends and family; life events; and hopelessness. Risk factors collected prior to the age 16‐year assessment included gender; IQ; executive function; impulsivity; personality dimensions; parent suicide attempt; sexual abuse; parental cruelty; being bullied; body dissatisfaction; psychiatric disorder; depression symptoms; heavy drinking; cannabis use; and illicit drug use (excluding cannabis).

#### Possible confounders

Additional analyses controlled for the possible confounding effects of child gender and socioeconomic position (SEP). We did not adjust for additional confounders, as our aim was to identify potential differences in risk factors for suicidal thoughts and attempts rather than to build the most parsimonious prediction model.

Socioeconomic position was assessed via maternal questionnaire and included (a) average weekly household disposable income recorded at age 3 and 4 years, divided into quintiles and rescaled to account for family size, composition and estimated housing benefits (Gregg, Propper, & Washbrook, 2008); (b) social class (professional/managerial or other, the highest of maternal or paternal social class was used) identified during pregnancy and (c) highest maternal educational attainment (less than O‐level, O‐level, A‐Level or university degree) measured during pregnancy (O‐levels and A‐levels are school qualifications taken around age 16 and 18 years respectively). The number of participants with complete data on suicidal outcomes and all included covariates was 4,097.

### Statistical analysis

Multinomial regression was used to examine associations between each individual risk factor and a three‐category outcome: no suicidal ideation or attempts, suicidal ideation only and suicide attempts. Models were adjusted for sex and SEP. To obtain a direct comparison between suicidal ideation and attempts, each model was re‐estimated with an alternative reference group to provide this additional information. A risk factor was considered to be associated with an outcome if the confidence interval did not include the null (*p* value <.05). Continuous risk factors were standardised before analysis to create *Z* scores with a mean of 0 and a *SD* of 1. We explored the possibility of nonlinearity by dividing continuous risk factors into quintiles and testing whether a categorical variable was a better fit to the data using the likelihood ratio test. Extraversion showed evidence of a ‘J‐shape’ relationship, and so this variable was split into three categories prior to analysis (lowest quintile, highest quintile, middle quintiles). All analyses were conducted using Stata version 14.

Primary analyses were conducted on an imputed data set based on those with complete data on suicidal ideation and attempts (*n* = 4,772) (see Appendix S1). Results of the primary analysis are presented in Table 2 and Table S3. A comparison of the estimates from the complete case and imputed data analysis is presented in Table S4a,b; effect estimates were broadly consistent in the imputed and complete case analysis.

**Table 2:** Multinomial logit model showing differences between those with suicidal thoughts and attempts. Results are based on imputed data

Risk factor	Suicide attempts versus suicidal thoughts only				
	Sample mean (*SD*) or % (*n*)^a^	Unadjusted	*p* Value	Adjusted for gender and SEP	*p* Value
Demographic variables					
Female gender (birth)	2,389 (58.3%)	1.58 (1.12, 2.24)	.010**	1.55 (1.10, 2.20)	.013*
Psychosocial variables					
Total IQ (8 years)	107.9 (16.1)	0.75 (0.64, 0.88)	<.001**	0.80 (0.67, 0.96)	.017*
Executive function (8 years)					
Updating	12.7 (3.9)	0.88 (0.75, 1.02)	.091	0.90 (0.77, 1.05)	.172
Attentional switching	11.0 (16.0)	1.06 (0.91, 1.23)	.447	1.05 (0.91, 1.22)	.488
Attentional control	16.5 (7.7)	0.95 (0.83, 1.09)	.476	0.95 (0.82, 1.10)	.491
Impulsivity (10 years)	13.7 (2.5)	1.19 (1.01, 1.41)	.041*	1.19 (1.01, 1.42)	.042*
Sensation seeking (16 years)					
Arnett intensity subscale	25.9 (4.6)	1.06 (0.92, 1.23)	.392	1.17 (1.00, 1.37)	.048*
Arnett novelty subscale	25.9 (4.3)	0.90 (0.78, 1.04)	.162	0.96 (0.83, 1.11)	.589
Big‐5 personality dimensions (14 years)					
Extraversion (first quintile: lowest)	672 (21.7%)	0.79 (0.53, 1.19)	.260	0.81 (0.54, 1.22)	.316
Extraversion (mid‐quintiles)	1,844 (59.6%)	–		–	
Extraversion (last quintile: highest)	580 (18.7%)	1.20 (0.76, 1.90)	.428	1.19 (0.75, 1.88)	.460
Agreeableness	38.3 (5.1)	0.89 (0.75, 1.07)	.212	0.87 (0.72, 1.06)	.175
Conscientiousness	32.0 (6.0)	0.84 (0.70, 0.99)	.037*	0.84 (0.71, 0.99)	.046*
Emotional stability	31.7 (6.5)	0.88 (0.74, 1.04)	.132	0.92 (0.77, 1.10)	.354
Intellect/openness to experience	36.1 (5.7)	0.84 (0.71, 0.99)	.042*	0.89 (0.74, 1.06)	.187
Self‐harm in friends and family					
Parent suicide attempt (birth to 11 years)	56 (1.5%)	1.90 (0.82, 4.39)	.131	1.65 (0.71, 3.84)	.247
Family self‐harm (16 years)	359 (8.8%)	2.15 (1.53, 3.01)	<.001**	1.95 (1.39, 2.75)	<.001**
Friend self‐harm (16 years)	1,614 (39.6%)	2.69 (1.93, 3.75)	<.001**	2.61 (1.85, 3.68)	<.001**
Extent of exposure (16 years)					
Either friend or family member self‐harm (one only)	1,523 (37.5%)	3.28 [2.20, 4.87]	<.001**	3.21 [2.14, 4.82]	<.001**
Both friend and family member self‐harm	221 (5.5%)	5.66 [3.45, 9.28]	<.001**	5.26 [3.17, 8.74]	<.001**
Number of life events (8 years)	3.0 (2.1)	1.22 (1.08, 1.37)	.002**	1.18 (1.04, 1.33)	.011*
Childhood sexual abuse (birth to 8 years)	20 (0.5%)	1.58 (0.36, 6.90)	.544	1.30 (0.29, 5.71)	.732
Cruelty to children in household (birth to 11 years)	135 (4.5%)	1.63 (0.90, 2.96)	.109	1.71 (0.93, 3.11)	.082
Being bullied (12 years)	842 (25.5%)	1.24 (0.90, 1.71)	.181	1.26 (0.91, 1.73)	.162
Body dissatisfaction (13 years)	1,140 (32.8%)	1.80 (1.32, 2.45)	<.001**	1.70 (1.24, 2.34)	.001**
Psychiatric/mental health variables					
DAWBA diagnosis (15 years)					
Depressive disorder	47 (1.6%)	3.79 (1.77, 8.14)	.001**	3.63 (1.67, 7.89)	.001**
Anxiety disorder	50 (1.7%)	2.34 (1.20, 4.56)	.012*	2.20 (1.12, 4.30)	.022*
Behavioural disorder (ODD/CD/ADHD)	91 (3.3%)	2.90 (1.55, 5.42)	.001**	2.90 (1.54, 5.44)	.001**
Depressive symptoms (12 years)	3.9 (3.8)	1.10 (0.98, 1.23)	.104	1.09 (0.97, 1.22)	.157
Hopelessness (16 years)	650 (16.6%)	1.52 (1.13, 2.03)	.005**	1.47 (1.10, 1.98)	.010*
Substance use (15 years)					
Alcohol (heavy drinking)	539 (18.6%)	1.31 (0.98, 1.74)	.232	1.23 (0.82, 1.84)	.321
Cannabis (occasional)	246 (8.3%)	1.13 (0.69, 1.85)	.633	1.15 (0.70, 1.89)	.578
Smoking (weekly)	217 (8.6%)	2.74 (1.75, 4.31)	<.001**	2.54 (1.61, 4.02)	<.001**
Other illicit drug use (past year)^b^	321 (11.4%)	1.80 (1.19, 2.74)	.006**	1.80 (1.18, 2.75)	.006**

^a^Percentage/mean in the whole sample. Numbers vary due to missing data.

^b^Other illicit drug use does not include cannabis.

The results in Table 2 were generated by re‐estimating each model with an alternative reference group.

Continuous risk factors are standardised (*Z* scores).

*p <* *.05; **p <* *.01.

## Results

Of the 4,772 participants with complete outcome data at age 16 years, 3,991 (83.6%) reported no suicidal ideation or attempts, 456 (9.6%) reported suicidal ideation only and 325 (6.8%) reported a suicide attempt. Descriptive information on risk factors according to outcome group is provided in Table S2. Those with a history of suicidal ideation or attempts generally had higher levels of risk factors than those without. Risk factor levels were also generally higher amongst those who had made a suicide attempt than those who had only thought about suicide.

Findings from the multinomial logistic regression analyses are shown in Table 2 and Table S3. These analyses are based on an imputed data set (*N* = 4,772). The ORs in Table S3 indicate the likelihood of membership in the suicidal ideation group and the suicide attempt group relative to the group without suicidal ideation or attempts. Most of the risk factors we investigated were associated with either suicidal ideation, suicide attempts or both outcomes when compared to those without any suicidal ideation or attempts (Table S3). The ORs in Table 2 indicate the likelihood that individuals with suicidal ideation also attempted suicide.

### Risk factors that differentiate between those with a history of suicidal ideation and those who have made an attempt

The results for each risk factor are shown in Table 2. The factors that most clearly differentiated between those with a history of ideation and attempts at age 16 years were depressive disorder (OR 3.63 [95% CI: 1.67, 7.89]), behavioural disorder (OR 2.90 [95% CI: 1.54, 5.44]), anxiety disorder (OR 2.20 [95% CI: 1.12, 4.30]), exposure to self‐harm in others (either family/friend self‐harm OR 3.21 [95% CI: 2.14, 4.82]; both friend and family self‐harm OR 5.26 [95% CI: 3.17, 8.74]) and smoking [OR 2.54 [95% CI: 1.61, 4.02]. Other risk factors that were more strongly associated with suicide attempts compared to ideation included female gender, lower IQ, higher intensity seeking, lower conscientiousness, a greater number of life events, body dissatisfaction, hopelessness and illicit drug use (excluding cannabis).

## Discussion

This study investigated factors distinguishing between adolescents who had thought about suicide and those who had made an attempt. Over half of the risk factors we considered distinguished between those with ideation and attempts, however, many had small effects. Notably larger effect sizes were found for exposure to self‐harm in family/friends, mental health disorders and also smoking and illicit drug use (excluding cannabis).

Recent theoretical models all emphasise the role of ‘suicide capability’ in the progression from suicidal ideation to attempts (Klonsky & May, 2015; O'Connor, 2011; Van Orden et al., 2010). This capability is thought to be developed and enhanced through exposure to painful and provocative events, which lead to an increased tolerance to pain, fear and death. Our findings are consistent with this idea, as one of the factors which most clearly differentiated between ideation and attempts – exposure to self‐harm in friends/family – may serve to increase the acquired capability for suicide. Another factor thought to increase suicide capability is non‐suicidal self‐harm; however, it was not possible to identify whether participants in this study who reported a suicide attempt had also engaged in non‐suicidal self‐harm.

Compared to ideators, those who acted on their thoughts were more likely to have been exposed to self‐harm in others. Moreover, there was evidence of a dose–response effect, as associations were notably larger when participants were exposed to self‐harm in both friends and family [OR 5.26]. Previous research has shown family history to be a risk factor for suicidal behaviour, over and above the risk associated with psychiatric disorder, and exposure has been shown to differentiate between suicidal ideation and attempts in several other studies (Asarnow et al., 2008; Dhingra et al., 2015; O'Connor et al., 2012). The potential mechanisms underlying this relationship require further study but could include genetic influences, imitation, social transmission and assortative relating. In contrast to these findings, a recent study of hospitalised adolescents found no association between family history of suicidal behaviour and suicidal ideation/behaviours (Goldston et al., 2016). Our sample was population‐based, and we were not able to explore associations with hospitalisation/service use. Future research is needed to explore whether those who are hospitalised for mental health problems are more likely to be exposed to self‐harm as this would have important implications for treatment.

Other risk factors that distinguished suicide attempters from ideators were smoking and illicit drug use (excluding cannabis). In contrast, heavy drinking and cannabis use did not differ across these groups, highlighting the importance of examining substances individually. Alcohol is the substance that has been researched most widely in the literature, as it is thought that its intoxicating effects may increase aggression, impair decision‐making and lower inhibition, making it more likely that someone will act on suicidal thoughts. However, most previous studies, including a recent meta‐analysis of adults, have not found alcohol problems to be elevated amongst attempters compared to ideators (Borges et al., 2008; ten Have, van Dorsselaer, & de Graaf, 2013; May & Klonsky, 2016; Nock et al., 2009; Taliaferro & Muehlenkamp, 2014). The same meta‐analysis reported a moderate effect for drug use disorders (May & Klonsky, 2016), which is consistent with our findings. It may also be that substance use is a proxy for particular types of coping in response to stress which are maladaptive.

Strong associations have previously been reported between smoking and a range of suicide‐related outcomes, even after adjustment for potential confounders, such as depression (Bronisch, Höfler, & Lieb, 2008; King et al., 2001). However, few studies have examined smoking within an ‘ideation to action’ framework. A cross‐sectional study found suicide attempters were more likely than ideators to be current smokers (King et al., 2001). However, in the National Comorbidity Survey, Kessler, Borges, Sampson, Miller, and Nock (2009) found that early‐onset nicotine dependence was prospectively associated with suicide plans but not attempts amongst those with ideation.

With regard to mental health disorders, previous studies of adolescents suggest that depression/dysthymia and disorders characterised by agitation and poor impulse control best distinguish between those with ideation and attempts, as found in this study (Nock et al., 2013). However, like for many risk factors, findings have been conflicting. Although depressive disorder at age 15 years clearly distinguished between those with ideation and attempts in this sample, it was relatively uncommon (2.6% of ideators and 8.3% of attempters) and so would have limited sensitivity as a predictor.

Other variables that have been highlighted in the literature include hopelessness and impulsivity. Whilst we found both these factors differentiated between thoughts and attempts, effects were relatively small, indicating that other factors may be more useful at distinguishing between these groups. In addition, given the debate about how impulsivity is operationalised (Gvion & Apter, 2011) and that the assessment of hopelessness was limited to two items, future research should explore the respective roles of these factors in the context of ideation to action in more detail.

### Strengths and limitations

This study offers many improvements over previous work. Data were from a large population‐based birth cohort containing information on suicidal ideation and attempts from over 4,000 adolescents. This is important as most episodes of suicidal behaviour do not present to specialist services (Kidger et al., 2012). We also explored a wide range of risk factors from multiple domains. However, findings need to be interpreted in light of several limitations. First, whereas ALSPAC is a longitudinal study, lifetime history of suicidal ideation and attempts were both assessed at the same time point, when participants were aged 16 years. These outcomes were assessed via self‐report, which may be subject to misreporting (Mars et al., 2016). For example, we have previously shown that more recent and more severe episodes of self‐harm are more likely to be reported consistently over time (Mars et al., 2016). Suicidal thoughts are less severe than suicide attempts and may be more easily forgotten. This may explain why our ratio of ideation to attempts is lower than found in some previous studies (Nock et al., 2013; O'Connor & Nock, 2014; Taliaferro & Muehlenkamp, 2014). Moreover, our measure of suicidal ideation was assessed with a single item, which does not allow us to establish severity. It is possible that findings may differ for more ‘active’ rather than passive suicidal ideation.

Second, the age of onset of suicidal ideation and attempts was not known; therefore, for some variables, (particularly those measured close to age 16) reverse causation is possible. We found that associations tended to be stronger for those variables measured more proximally to the outcome (e.g. exposure to self‐harm, psychiatric disorders), although this was not always the case (e.g. there was little evidence of an association for alcohol or cannabis use). Moreover, for some variables (gender, IQ, executive functioning, personality), the timing of assessment would have little or no impact on the results, as these variables are fairly stable or fixed over time. As the study involved secondary analysis of an existing data set, the selection of measures was dependent on data availability, and we were not able to test hypotheses regarding the timing of exposure, or whether the findings would differ according to developmental stage.

Third, we only adjusted for a limited number of confounding variables (gender and SEP) and it is possible that there may be residual confounding. However, it was not our aim to identify independent predictors and to examine this adequately would require a separate theory‐driven analytical model for each exposure. This was beyond the scope of the current paper but is an important area for future research. We did not correct for multiple testing as analyses were exploratory, and a number of risk factors are likely highly correlated. Our results are therefore in need of replication, given the large number of tests conducted.

Fourth, analyses were conducted on an imputed data set based on those with complete outcome data at age 16 years (*N* = 4,772). Our imputation analysis assumes follow‐up depends only on those individual characteristics observed and included in the imputation model (see Appendix S1). If this assumption (the missing at random, MAR, assumption) is not true, then these results may be biased. Moreover, responders and non‐responders to the self‐harm questionnaire differed on a range of characteristics (see Table S1), and it is possible that this non‐random non‐response may limit the generalisability of our results.

Finally, our study sample was a population‐based sample of 16‐year‐old adolescents, and findings may not generalise to fatal attempts, clinical samples or to other age groups (including younger adolescents). It is likely that the risk factors involved in progression from ideation to attempts may vary across the life course. This possibility should be investigated in future research.

### Conclusion and clinical implications

The results of this study have important implications for both clinical practice and suicide theory. Identifying factors that differentiate between those with suicidal ideation and attempts can help to improve risk assessment (when treating individuals with suicidal ideation) and identify potential targets for intervention. Our findings suggest that youths who make suicide attempts are likely to have had exposure to self‐harm in others, underscoring the importance of practitioners considering the social context in which suicide attempts occur and the possibility of modelling, contagion and environmental reinforces of self‐harm behaviour**.** Our results also highlight the continued importance of identifying and treating mental health problems, as they are strongly associated with the development of suicidal ideation and likely have a role in progression from thoughts to behaviour. It is also important for suicide prevention efforts to consider disorders other than depression. Future research should build on these findings and prospectively follow‐up those with suicidal ideation, to explore whether the factors identified in this study predict subsequent suicide risk.

**Key points**

- A third of young people who experience suicidal ideation attempt suicide. However, little is known about the factors that differentiate those most likely to attempt suicide from those who only think about suicide.
- We examined this issue in a population‐based sample of over 4,700 adolescents.
- The factors that most clearly differentiated between those with a history of suicidal ideation and attempts were exposure to self‐harm in friends/family and psychiatric disorder.
- Prospective studies are needed to explore whether these risk factors predict future suicide attempts.

## Supporting information


**Table S1.** Comparison of responders and non‐responders to the self‐harm questionnaire at 16 years by key demographic variables.
**Table S2.** Descriptive table for risk factors.
**Table S3.** Multinomial logit model investigating associations between risk factors and adolescent suicidal thoughts and attempts.
**Table S4a.** Comparison of complete case and imputed analysis.
**Table S4b.** Comparison of complete case and imputed analysis.
**Appendix S1.** Missing data.
