# Supplementary material for: What distinguishes adolescents with suicidal thoughts from those who have attempted suicide? A population‐based birth cohort study
Source: J Child Psychol Psychiatry. 2018 Mar 1;60(1):91–9. doi: 10.1111/jcpp.12878 (PMC6334515; doi:10.1111/jcpp.12878)
Supplement: Supplementary file 1 — Table S1. Comparison of responders and non‐responders to the self‐harm questionnaire at 16 years by key demographic variables. Table S2. Descriptive table for risk factors. Table S3. Multinomial logit model investigating associations between risk factors and adolescent suicidal thoughts and attempts. Table S4a. Comparison of complete case and imputed analysis. Table S4b. Comparison of complete case and imputed analysis. Appendix S1. Missing data. [file JCPP-60-91-s001.docx]

**Table S1:** Comparison of responders and non-responders to the self-harm questionnaire at 16 years by key demographic variables (Comparison amongst those invited to participate in the age 16 year questionnaire, *N*=9,370)

| Variable | Description | *N* | No data on suicidal thoughts and attempts ^a^ | Data on suicidal thoughts and attempts | χ^2^ | *P* value |
| --- | --- | --- | --- | --- | --- | --- |
| Child gender | Male | 4,628 | 2,672 (58.1%) | 1,956 (41.0%) | 274.7 | <0.001 |
|  | Female | 4,742 | 1,956 (41.9%) | 2,816 (59.0%) |  |  |
| Parity | First born | 4,060 | 1,789 (41.5%) | 2,271 (48.8%) | 67.8 | <0.001 |
|  | Second born | 3,199 | 1,571 (36.5%) | 1,628 (35.0%) |  |  |
|  | Third born plus | 1,700 | 948 (22.0%) | 752 (16.2%) |  |  |
| Home overcrowding | <1 person/room | 8,434 | 3,994 (93.1%) | 4,440 (96.6%) | 55.7 | <0.001 |
|  | >1 person/room | 453 | 296 (6.9%) | 157 (3.4%) |  |  |
| Maternal education | Degree | 1,306 | 365 (8.7%) | 941 (20.2%) | 424.7 | <0.001 |
|  | A level | 2,206 | 906 (21.5%) | 1,300 (28.0%) |  |  |
|  | O level | 3,106 | 1,561 (37.0%) | 1,545 (33.2%) |  |  |
|  | < O level | 2,246 | 1,383 (32.8%) | 863 (18.6%) |  |  |
| Equivalised household income | 1^st^ quintile | 1,724 | 599 (16.7%) | 1,125 (25.9%) | 205.9 | <0.001 |
|  | 2^nd^ quintile | 1,657 | 651 (18.2%) | 1,006 (23.1%) |  |  |
|  | 3^rd^ quintile | 1,612 | 753 (21.0%) | 859 (19.8%) |  |  |
|  | 4^th^ quintile | 1,519 | 762 (21.3%) | 757 (17.4%) |  |  |
|  | 5^th^ quintile | 1,417 | 818 (22.8%) | 599 (13.8%) |  |  |
| Parental social class | Professional/managerial | 4,870 | 1,979 (50.6%) | 2,891 (64.9%) | 174.5 | <0.001 |
|  | other | 3,499 | 1,933 (49.4%) | 1,566 (35.1%) |  |  |
| Child ethnicity | White | 8,304 | 3,898 (94.8%) | 4,406 (96.1%) | 9.7 | 0.002 |
|  | Non-white | 393 | 216 (5.2%) | 177 (3.9%) |  |  |

*^a^ Includes those who were sent but did not return the self-harm questionnaire, and those who returned the questionnaire but did not respond to the suicidal thoughts or attempts items*

**Table S2:** Descriptive table for risk factors

| **Exposure (age of assessment)** | **No suicidal thoughts or attempts (3,991)** | | **Suicidal thoughts only (n=456)** | | **Suicide attempts (n=325)** | | **P value*** |
| --- | --- | --- | --- | --- | --- | --- | --- |
| **Demographic and socioeconomic variables** |  |  |  |  |  |  |  |
| Female gender, n (%) | 2,218 | (55.6%) | 334 | (73.3%) | 264 | (81.2%) | <0.001 |
|  |  |  |  |  |  |  |  |
| Equivalised income (33 & 47 months), n (%) ^a^ |  |  |  |  |  |  | 0.004 |
| 5^th^ quintile (lowest) | 490 | (13.5%) | 59 | (14.2%) | 50 | (16.9%) |  |
| 4^th^ quintile | 612 | (16.8%) | 71 | (17.1%) | 74 | (25.0%) |  |
| 3^rd^ quintile | 712 | (19.6%) | 89 | (21.4%) | 58 | (19.6%) |  |
| 2^nd^ quintile | 852 | (23.5%) | 93 | (22.4%) | 61 | (20.6%) |  |
| 1^st^ quintile (highest) | 968 | (26.6%) | 104 | (25.0%) | 53 | (17.9%) |  |
|  |  |  |  |  |  |  |  |
| Parent social class (pregnancy), n (%) ^b^ |  |  |  |  |  |  |  |
| *Other* | 1,285 | (34.4%) | 151 | (35.3%) | 130 | (44.2%) | 0.003 |
| *Professional/managerial* | 2,450 | (65.6%) | 277 | (64.7%) | 164 | (55.8%) |  |
|  |  |  |  |  |  |  |  |
| Mother’s education (pregnancy), n (%) |  |  |  |  |  |  | 0.080 |
| *<O-level* | 714 | (18.4%) | 79 | (17.8%) | 70 | (22.3%) |  |
| *O-level* | 1,283 | (33.0%) | 150 | (33.8%) | 112 | (35.7%) |  |
| *A level* | 1,089 | (28.0%) | 121 | (27.3%) | 90 | (28.7%) |  |
| *Degree* | 805 | (20.7%) | 94 | (21.2%) | 42 | (13.4%) |  |
|  |  |  |  |  |  |  |  |
| **Psychosocial variables** |  |  |  |  |  |  |  |
| Total IQ (age 8), mean (SD) | 107.57 | (16.12) | 108.83 | (16.15) | 105.05 | (16.56) | 0.018 |
|  |  |  |  |  |  |  |  |
| Executive function (age 8), mean (SD) |  |  |  |  |  |  |  |
| *Updating* | 12.72 | (3.89) | 12.85 | (3.66) | 12.38 | (3.84) | 0.316 |
| *Attentional-switching* | 10.87 | (15.81) | 10.64 | (14.45) | 11.77 | (21.77) | 0.659 |
| *Attentional-control* | 16.40 | (5.94) | 17.34 | (15.77) | 16.61 | (5.51) | 0.075 |
|  |  |  |  |  |  |  |  |
| Impulsivity (age 10), stop-signal task, mean number of trials correct at 250ms delay (SD) | 13.69 | (2.58) | 13.91 | (2.39) | 13.49 | (2.72) | 0.133 |
|  |  |  |  |  |  |  |  |
| Sensation-seeking (age 16), mean (SD) |  |  |  |  |  |  |  |
| *Arnett intensity subscale* | 25.77 | (4.51) | 25.95 | (4.78) | 26.24 | (4.51) | 0.165 |
| *Arnett novelty subscale* | 25.79 | (4.26) | 26.11 | (4.51) | 25.65 | (4.72) | 0.266 |
|  |  |  |  |  |  |  |  |
| Big-5 personality dimensions (age 14) |  |  |  |  |  |  |  |
| *Extraversion (first quintile: lowest), n (%)* | 613 | (20.7%) | 100 | (37.8%) | 55 | (24.7%) | <0.001 |
| *Extraversion (mid quintiles), n (%)* | 1794 | (60.5%) | 163 | (51.8%) | 120 | (53.8%) |  |
| *Extraversion (last quintile: highest), n (%)* | 557 | (18.8%) | 52 | (16.5%) | 48 | (21.5%) |  |
| *Agreeableness*, mean (SD) | 38.09 | (5.15) | 39.30 | (4.91) | 38.79 | (5.07) | <0.001 |
| *Conscientiousness*, mean (SD) | 32.27 | (5.93) | 31.14 | (6.01) | 29.99 | (5.96) | <0.001 |
| *Emotional stability*, mean (SD) | 32.39 | (6.25) | 28.03 | (6.43) | 27.61 | (6.96) | <0.001 |
| *Intellect/ openness to experience*, mean (SD) | 35.94 | (5.67) | 36.81 | (5.63) | 36.10 | (6.39) | 0.035 |
|  |  |  |  |  |  |  |  |
| Self-harm in friends and family, n (%) |  |  |  |  |  |  |  |
| *Parent suicide attempt (birth-age 11)* | 40 | (1.1%) | 8 | (2.1%) | 14 | (4.9%) | <0.001 |
| *Family self-harm (age 16) ^c^* | 264 | (6.7%) | 80 | (17.5%) | 101 | (31.4%) | <0.001 |
| *Self-harm in friends (age 16) ^c^* | 1,345 | (33.9%) | 276 | (60.7%) | 261 | (80.6%) | <0.001 |
|  |  |  |  |  |  |  |  |
| Extent of exposure  *None*  *Friend/family only*  *Both* | 2,488  1,318  142 | (63.0%)  (33.4%)  (3.6%) | 153  248  54 | (33.6%)  (54.5%)  (11.9%) | 39  205  77 | (12.2%)  (63.7%)  (24.0%) | <0.001 |
|  |  |  |  |  |  |  |  |
| Life events (age 16), mean (SD) | 2.81 | (1.99) | 3.92 | (2.28) | 4.49 | (2.61) | <0.001 |
| Sexual abuse (birth - age 8), *n (%)* | ^d^ |  | ^d^ |  | ^d^ |  | 0.146 |
| Parental cruelty to children (birth- age 11), *n (%)* | 100 | (3.7%) | 20 | (6.9%) | 24 | (11.4%) | <0.001 |
| Being Bullied (age 12), *n (%)* | 720 | (22.8%) | 130 | (36.5%) | 97 | (40.3%) | <0.001 |
| Body dissatisfaction (age 13), n *(%)* | 988 | (29.7%) | 159 | (42.5%) | 153 | (56.9%) | <0.001 |
|  |  |  |  |  |  |  |  |
| **Psychiatric/mental health variables** |  |  |  |  |  |  |  |
| DAWBA diagnosis (age 15), n (%) |  |  |  |  |  |  |  |
| *Depressive disorder* | 26 | (0.9%) | 8 | (2.6%) | 18 | (8.3%) | <0.001 |
| *Anxiety disorder* | 25 | (0.9%) | 14 | (4.4%) | 18 | (8.3%) | <0.001 |
| *Behavioural disorder (ODD/CD/ADHD)* | 58 | (2.2%) | 16 | (5.6%) | 29 | (14.1%) | <0.001 |
|  |  |  |  |  |  |  |  |
| Depressive symptoms (age 12) , mean (SD) | 3.54 | (3.40) | 6.05 | (4.77) | 6.44 | (5.10) | <0.001 |
| Hopelessness (age 16), n (%) | 423 | (11.1%) | 184 | (42.4%) | 154 | (51.9%) | <0.001 |
|  |  |  |  |  |  |  |  |
| Substance use (age 15), n (%) |  |  |  |  |  |  |  |
| *Alcohol, heavy drinking* | 502 | (18.0%) | 66 | (21.8%) | 54 | (25.7%) | 0.008 |
| *Cannabis, at least occasional use* | 207 | (7.2%) | 39 | (12.5%) | 31 | (14.4%) | <0.001 |
| *Smoking, at least weekly* | 176 | (7.2%) | 31 | (11.7%) | 52 | (28.3%) | <0.001 |
| *Other illicit drug use (post year) ^e^* | 279 | (10.3%) | 49 | (16.5%) | 54 | (26.9%) | <0.001 |

** Chi-Square test of the association between self-harm and categorical exposures and ANOVA for differences in means for continuous exposures*

*^a^ Quintiles represent lowest to highest household income. Quintiles were derived from income measures at ages 33 and 47 months on a larger subset of the cohort, and so in the present sample numbers are not evenly distributed*

*^b^ Highest social class of mother and father*

*^c^ Child-rated self-harm exposure*

*^d^ Numbers for sexual abuse cannot be shown due to the small cell count^e^ Other illicit drug use does not include cannabis*

**Table S3:** Multinomial logit model investigating associations between risk factors and adolescent suicidal thoughts and attempts. Results are based on imputed data (n=4,772)

| **Risk factor** | **Suicidal thoughts only versus no suicidal thoughts or attempts** | | | | **Suicide attempts versus no suicidal thoughts or attempts** | | | |
| --- | --- | --- | --- | --- | --- | --- | --- | --- |
|  | **Unadjusted** | **P value** | **Adjusted for gender and SEP** | **P value** | **Unadjusted** | **P value** | **Adjusted for gender and SEP** | **P value** |
| **Demographic variables** |  |  |  |  |  |  |  |  |
| Female gender | 2.19 (1.76, 2.72) | <0.001** | 2.20 (1.77, 2.73) | <0.001** | 3.46 (2.60, 4.60) | <0.001* | 3.41 (2.56, 4.55) | <0.001** |
| **Psychosocial variables** |  |  |  |  |  |  |  |  |
| Total IQ | 1.09 (0.98, 1.21) | 0.127 | 1.14 (1.01, 1.29) | 0.031* | 0.82 (0.72, 0.93) | 0.002** | 0.92 (0.80, 1.06) | 0.246 |
|  |  |  |  |  |  |  |  |  |
| Executive function |  |  |  |  |  |  |  |  |
| *Updating* | 1.03 (0.93, 1.15) | 0.563 | 1.02 (0.91, 1.14) | 0.792 | 0.91 (0.80, 1.02) | 0.111 | 0.91 (0.80, 1.03) | 0.148 |
| *Attentional-switching* | 1.00 (0.89, 1.12) | 0.995 | 1.02 (0.91, 1.15) | 0.715 | 1.06 (0.95, 1.18) | 0.290 | 1.08 (0.96, 1.20) | 0.187 |
| *Attentional-control* | 1.09 (0.99, 1.19) | 0.066 | 1.09 (0.99, 1.19) | 0.073 | 1.04 (0.91, 1.18) | 0.602 | 1.03 (0.90, 1.19) | 0.646 |
|  |  |  |  |  |  |  |  |  |
| Impulsivity | 0.91 (0.81, 1.03) | 0.155 | 0.92 (0.81, 1.04) | 0.170 | 1.09 (0.96, 1.24) | 0.167 | 1.09 (0.96, 1.24) | 0.161 |
|  |  |  |  |  |  |  |  |  |
| Sensation-seeking |  |  |  |  |  |  |  |  |
| *Arnett intensity subscale* | 1.04 (0.95, 1.15) | 0.392 | 1.24 (1.11, 1.38) | <0.001** | 1.11 (0.99, 1.24) | 0.070 | 1.45 (1.28, 1.65) | <0.001** |
| *Arnett novelty subscale* | 1.08 (0.98, 1.19) | 0.130 | 1.15 (1.03, 1.27) | 0.009** | 0.97 (0.87, 1.09) | 0.647 | 1.10 (0.98, 1.24) | 0.116 |
|  |  |  |  |  |  |  |  |  |
| Big-5 personality dimensions |  |  |  |  |  |  |  |  |
| *Extraversion (first quintile: lowest)* | 1.69 (1.30, 2.18) | <0.001** | 1.77 (1.36, 2.30) | <0.001** | 1.34 (0.97, 1.85) | 0.074 | 1.44 (1.04, 1.99) | 0.030* |
| *Extraversion (mid quintiles)* | - |  | - |  | - |  | - |  |
| *Extraversion (last quintile: highest)* | 0.95 (0.69, 1.31) | 0.766 | 0.88 (0.64, 1.22) | 0.448 | 1.15 (0.82, 1.61) | 0.427 | 1.05 (0.75, 1.48) | 0.776 |
| *Agreeableness* | 1.28 (1.13, 1.44) | <0.001** | 1.17 (1.03, 1.33) | 0.016* | 1.14 (0.99, 1.31) | 0.068 | 1.02 (0.88, 1.19) | 0.789 |
| *Conscientiousness* | 0.82 (0.73, 0.92) | 0.001** | 0.84 (0.74, 0.94) | 0.003** | 0.68 (0.60, 0.78) | <0.001** | 0.70 (0.62, 0.80) | <0.001** |
| *Emotional stability* | 0.50 (0.44, 0.56) | <0.001** | 0.52 (0.47, 0.59) | <0.001** | 0.44 (0.38, 0.50) | <0.001** | 0.48 (0.42, 0.56) | <0.001** |
| *Intellect/openness to experience* | 1.18 (1.06, 1.33) | 0.004** | 1.24 (1.10, 1.40) | <0.001** | 0.99 (0.87, 1.13) | 0.913 | 1.10 (0.96, 1.27) | 0.178 |
|  |  |  |  |  |  |  |  |  |
| Self-harm in friends and family |  |  |  |  |  |  |  |  |
| *Parent suicide attempt* | 2.37 (1.17, 4.78) | 0.016* | 2.37 (1.15, 4.87) | 0.019* | 4.50 (2.43, 8.36)) | <0.001** | 3.90 (2.05, 7.42) | <0.001** |
| *Family self-harm (child-rated)* | 2.99 (2.28, 3.92) | <0.001** | 2.70 (2.05, 3.56) | <0.001** | 6.43 (4.93, 8.39) | <0.001** | 5.27 (4.01, 6.93) | <0.001** |
| *Friend self-harm (child-rated)* | 3.01 (2.47, 3.68) | <0.001** | 2.63 (2.14, 3.24) | <0.001** | 8.10 (6.10, 10.7) | <0.001** | 6.86 (5.13, 9.19) | <0.001** |
| Extent of exposure  *Friend/family only*  *Both* | 3.07 [2.48, 3.79]  6.21 [4.36, 8.84] | <0.001**  <0.001** | 2.72 (2.18, 3.39)  5.34 (3.72, 7.65) | <0.001**  <0.001** | 10.0 [7.09, 14.2]  35.2 [23.1, 53.5] | <0.001**  <0.001** | 8.73 (6.11, 12.5)  28.1 (18.2, 43.2) | <0.001**  <0.001** |
|  |  |  |  |  |  |  |  |  |
| Number of life events | 1.65 (1.50, 1.80) | <0.001** | 1.61 (1.47, 1.77) | <0.001** | 2.00 (1.80, 2.22) | <0.001** | 1.89 (1.70, 2.10) | <0.001** |
| Childhood sexual abuse | 1.59 (0.50, 5.13) | 0.434 | 1.44 (0.44, 4.71) | 0.544 | 2.52 (0.85, 7.45) | 0.095 | 1.87 (0.62, 5.64) | 0.268 |
| Cruelty to children in household | 2.01 (1.25, 3.24) | 0.004** | 2.08 (1.29, 3.37) | 0.003** | 3.28 (2.10, 5.12) | <0.001** | 3.55 (2.26, 5.58) | <0.001** |
| Being Bullied | 1.93 (1.54, 2.43) | <0.001** | 1.99 (1.58, 2.51) | <0.001** | 2.40 (1.85, 3.12) | <0.001** | 2.50 (1.92, 3.27) | <0.001** |
| Body dissatisfaction | 1.77 (1.42, 2.20) | <0.001** | 1.57 (1.26, 1.96) | <0.001** | 3.18 (2.48, 4.07) | <0.001** | 2.67 (2.07, 3.44) | <0.001** |
|  |  |  |  |  |  |  |  |  |
| **Psychiatric/mental health variables** |  |  |  |  |  |  |  |  |
| DAWBA diagnosis |  |  |  |  |  |  |  |  |
| *Depressive disorder* | 2.67 (1.22, 5.84) | 0.014* | 2.33 (1.06, 5.14) | 0.036* | 10.1 (5.42, 18.9) | <0.001** | 8.46 (4.44, 16.1) | <0.001** |
| *Anxiety disorder* | 5.12 (2.76, 9.53) | <0.001** | 4.41 (2.34, 8.29) | <0.001** | 12.0 (6.67, 21.6) | <0.001** | 9.67 (5.27, 17.8) | <0.001** |
| *Behavioural disorder (ODD/CD/ADHD)* | 2.63 (1.56, 4.43) | <0.001** | 2.76 (1.63, 4.68) | <0.001** | 7.64 (4.93, 11.8) | <0.001** | 8.01 (5.07, 12.6) | <0.001** |
|  |  |  |  |  |  |  |  |  |
| Depressive symptoms | 1.72 (1.57, 1.88) | <0.001** | 1.68 (1.53, 1.84) | <0.001** | 1.89 (1.70, 2.09) | <0.001** | 1.82 (1.64, 2.02) | <0.001** |
| Hopelessness | 5.86 (4.74, 7.25) | <0.001** | 5.73 (4.62, 7.11) | <0.001** | 8.90 (6.97, 11.4) | <0.001** | 8.44 (6.58, 10.8) | <0.001** |
| Substance use |  |  |  |  |  |  |  |  |
| *Alcohol (heavy drinking)* | 1.31 (0.98, 1.74) | 0.064 | 1.26 (0.95, 1.67) | 0.115 | 1.67 (1.22, 2.29) | 0.001** | 1.54 (1.13, 2.12) | 0.007** |
| *Cannabis (occasional)* | 1.94 (1.35, 2.79) | <0.001** | 2.00 (1.39, 2.89) | <0.001** | 2.19 (1.46, 3.28) | <0.001** | 2.31 (1.53, 3.48) | <0.001** |
| *Smoking (weekly)* | 1.81 (1.26, 2.60) | 0.001** | 1.70 (1.18, 2.46) | 0.005** | 4.95 (3.52, 6.97) | <0.001** | 4.33 (3.05, 6.15) | <0.001** |
| *Other illicit drug use (past year) ^a^* | 1.86 (1.35, 2.57) | <0.001** | 1.85 (1.34, 2.54) | <0.001** | 3.36 (2.46, 4.59) | <0.001** | 3.33 (2.42, 4.58) | <0.001** |

*^a^ Other illicit drug use does not include cannabis; Continuous risk factors are standardised (Z scores); * P <0.05; ** P <0.01*

**Table S4a: Comparison of complete case and imputed analysis**

| **Risk factors** | **Suicidal thoughts only versus no suicidal thoughts or attempts** | | | | **Suicide attempts versus no suicidal thoughts or attempts** | | | |
| --- | --- | --- | --- | --- | --- | --- | --- | --- |
|  | **Complete case: Adjusted for gender and SEP** | **P value** | **Imputed data: Adjusted for gender and SEP** | **P value** | **Complete case: Adjusted for gender and SEP** | **P value** | **Imputed data: Adjusted for gender and SEP** | **P value** |
| **Demographic variables** |  |  |  |  |  |  |  |  |
| Female gender | 2.09 (1.66, 2.64) | <0.001** | 2.20 (1.77, 2.73) | <0.001** | 3.17 (2.33, 4.30) | <0.001** | 3.41 (2.56, 4.55) | <0.001** |
| **Psychosocial variables** |  |  |  |  |  |  |  |  |
| Total IQ | 1.15 (1.01, 1.32) | 0..035* | 1.14 (1.01, 1.29) | 0.031* | 1.00 (0.86, 1.17) | 0.978 | 0.92 (0.80, 1.06) | 0.246 |
|  |  |  |  |  |  |  |  |  |
| Executive function |  |  |  |  |  |  |  |  |
| *Updating* | 1.03 (0.91, 1.17) | 0.657 | 1.02 (0.91, 1.14) | 0.792 | 0.94 (0.81, 1.08) | 0.384 | 0.91 (0.80, 1.03) | 0.148 |
| *Attentional-switching* | 0.98 (0.85, 1.12) | 0.743 | 1.02 (0.91, 1.15) | 0.715 | 1.00 (0.87, 1.17) | 0.925 | 1.08 (0.96, 1.20) | 0.187 |
| *Attentional-control* | 1.06 (0.98, 1.14) | 0.133 | 1.09 (0.99, 1.19) | 0.073 | 1.02 (0.89, 1.16) | 0.807 | 1.03 (0.90, 1.19) | 0.646 |
|  |  |  |  |  |  |  |  |  |
| Impulsivity | 0.90 (0.78, 1.03) | 0.128 | 0.92 (0.81, 1.04) | 0.170 | 1.09 (0.94, 1.25) | 0.250 | 1.09 (0.96, 1.24) | 0.161 |
|  |  |  |  |  |  |  |  |  |
| Sensation-seeking |  |  |  |  |  |  |  |  |
| *Arnett intensity subscale* | 1.24 (1.10, 1.39) | <0.001** | 1.24 (1.11, 1.38) | <0.001** | 1.45 (1.26, 1.66) | <0.001** | 1.45 (1.28, 1.65) | <0.001** |
| *Arnett novelty subscale* | 1.13 (1.02, 1.26) | 0.025* | 1.15 (1.03, 1.27) | 0.009** | 1.10 (0.96, 1.25) | 0.157 | 1.10 (0.98, 1.24) | 0.116 |
|  |  |  |  |  |  |  |  |  |
| Big-5 personality dimensions |  |  |  |  |  |  |  |  |
| *Extraversion (first quintile: lowest), n (%)* | 1.93 (1.45, 2.57) | <0.001** | 1.77 (1.36, 2.30) | <0.001** | 1.44 (1.01, 2.06) | 0.045* | 1.44 (1.04, 1.99) | 0.030* |
| *Extraversion (mid quintiles), n (%)* | - |  | - |  | - |  | - |  |
| *Extraversion (last quintile: highest), n (%)* | 0.99 (0.70, 1.41) | 0.966 | 0.88 (0.64, 1.22) | 0.448 | 1.22 (0.84, 1.76) | 0.298 | 1.05 (0.75, 1.48) | 0.776 |
| *Agreeableness* | 1.17 (1.02, 1.34) | 0.027* | 1.17 (1.03, 1.33) | 0.016* | 1.07 (0.91, 1.25) | 0.441 | 1.02 (0.88, 1.19) | 0.789 |
| *Conscientiousness* | 0.83 (0.74, 0.94) | 0.004** | 0.84 (0.74, 0.94) | 0.003** | 0.69 (0.60, 0.79) | <0.001** | 0.70 (0.62, 0.80) | <0.001** |
| *Emotional stability* | 0.53 (0.46, 0.60) | <0.001** | 0.52 (0.47, 0.59) | <0.001** | 0.52 (0.44, 0.61) | <0.001** | 0.48 (0.42, 0.56) | <0.001** |
| *Intellect/openness to experience* | 1.19 (1.04, 1.35) | 0.010** | 1.24 (1.10, 1.40) | <0.001** | 1.12 (0.96, 1.30) | 0.149 | 1.10 (0.96, 1.27) | 0.178 |
|  |  |  |  |  |  |  |  |  |
| Self-harm in friends and family |  |  |  |  |  |  |  |  |
| *Parent suicide attempt* | 1.73 (0.76, 3.95) | 0.191 | 2.37 (1.15, 4.87) | 0.019* | 3.41 (1.68, 6.90) | 0.001** | 3.90 (2.05, 7.42) | <0.001** |
| *Family self-harm (child-rated)* | 2.77 (2.05, 3.74) | <0.001** | 2.70 (2.05, 3.56) | <0.001** | 4.84 (3.56, 6.58) | <0.001** | 5.27 (4.01, 6.93) | <0.001** |
| *Friend self-harm (child-rated)* | 2.54 (2.03, 3.17) | <0.001** | 2.63 (2.14, 3.24) | <0.001** | 7.64 (5.51, 10.6) | <0.001** | 6.86 (5.13, 9.19) | <0.001** |
| Extent of exposure  *Friend/family only*  *Both* | 2.57 [2.03, 3.25]  5.39 [3.65, 7.96] | <0.001**  <0.001** | 2.72 (2.18, 3.39)  5.34 (3.72, 7.65) | <0.001**  <0.001** | 9.19 [6.21, 13.6]  27.9 [17.2, 45.2] | <0.001**  <0.001** | 8.73 (6.11, 12.5)  28.1 (18.2, 43.2) | <0.001**  <0.001** |
|  |  |  |  |  |  |  |  |  |
| Number of life events | 1.66 (1.49, 1.83) | <0.001** | 1.61 (1.47, 1.77) | <0.001** | 1.87 (1.66, 2.10) | <0.001** | 1.89 (1.70, 2.10) | <0.001** |
| Childhood sexual abuse | 1.09 (0.24, 4.81) | 0.914 | 1.44 (0.44, 4.71) | 0.544 | 1.99 (0.56, 7.11) | 0.287 | 1.87 (0.62, 5.64) | 0.268 |
| Cruelty to children in household | 2.10 (1.27, 3.48) | 0.004** | 2.08 (1.29, 3.37) | 0.003** | 3.51 (2.10, 5.85) | <0.001** | 3.55 (2.26, 5.58) | <0.001** |
| Being Bullied | 2.00 (1.56, 2.57) | <0.001** | 1.99 (1.58, 2.51) | <0.001** | 2.32 (1.73, 3.11) | <0.001** | 2.50 (1.92, 3.27) | <0.001** |
| Body dissatisfaction | 1.60 (1.26, 2.03) | <0.001** | 1.57 (1.26, 1.96) | <0.001** | 2.64 (1.99, 3.48) | <0.001** | 2.67 (2.07, 3.44) | <0.001** |
|  |  |  |  |  |  |  |  |  |
| **Psychiatric/mental health variables** |  |  |  |  |  |  |  |  |
| DAWBA diagnosis |  |  |  |  |  |  |  |  |
| *Depressive disorder* | 2.75 (1.15, 6.55) | 0.023* | 2.33 (1.06, 5.14) | 0.036* | 10.2 (5.19, 19.9) | <0.001** | 8.46 (4.44, 16.1) | <0.001** |
| *Anxiety disorder* | 4.57 (2.22, 9.42) | <0.001** | 4.41 (2.34, 8.29) | <0.001** | 8.01 (4.05, 15.8) | <0.001** | 9.67 (5.27, 17.8) | <0.001** |
| *Behavioural disorder (ODD/CD/ADHD)* | 2.91 (1.57, 5.40) | 0.001** | 2.76 (1.63, 4.68) | <0.001** | 8.31 (4.97, 13.9) | <0.001** | 8.01 (5.07, 12.6) | <0.001** |
|  |  |  |  |  |  |  |  |  |
| Depressive symptoms | 1.71 (1.55, 1.89) | <0.001** | 1.68 (1.53, 1.84) | <0.001** | 1.71 (1.53, 1.92) | <0.001** | 1.82 (1.64, 2.02) | <0.001** |
| Hopelessness | 5.78 (4.57, 7.30) | <0.001** | 5.73 (4.62, 7.11) | <0.001** | 8.02 (6.08, 10.6) | <0.001** | 8.44 (6.58, 10.8) | <0.001** |
|  |  |  |  |  |  |  |  |  |
| Substance use |  |  |  |  |  |  |  |  |
| *Alcohol (heavy drinking)* | 1.28 (0.93, 1.74) | 0.125 | 1.26 (0.95, 1.67) | 0.115 | 1.49 (1.05, 2.11) | 0.026* | 1.54 (1.13, 2.12) | 0.007** |
| *Cannabis (occasional)* | 2.00 (1.35, 2.95) | 0.001** | 2.00 (1.39, 2.89) | <0.001** | 2.47 (1.60, 3.82) | <0.001** | 2.31 (1.53, 3.48) | <0.001** |
| *Smoking (weekly)* | 1.86 (1.20, 2.87) | <0.001** | 1.70 (1.18, 2.46) | 0.005** | 4.34 (2.93, 6.45) | <0.001** | 4.33 (3.05, 6.15) | <0.001** |
| *Other illicit drug use (past year)* | 1.76 (1.22, 2.53) | 0.002** | 1.85 (1.34, 2.54) | <0.001** | 3.40 (2.35, 4.91) | <0.001** | 3.33 (2.42, 4.58) | <0.001** |

*Other illicit drug use does not include cannabis; Continuous risk factors are standardised (Z scores); * P <0.05; ** P <0.01***Table S4b: Comparison of complete case and imputed analysis**

|  | **Suicide attempts versus suicidal thoughts only** | | | |
| --- | --- | --- | --- | --- |
| **Risk factors** | **Complete case: Adjusted for gender and SEP** | **P value** | **Imputed data: Adjusted for gender and SEP** | **P value** |
| **Demographic variables** |  |  |  |  |
| Female gender | 1.51 (1.04, 2.19) | 0.029* | 1.55 (1.10, 2.20) | 0.013* |
| **Psychosocial variables** |  |  |  |  |
| Total IQ | 0.87 (0.71, 1.06) | 0.160 | 0.80 (0.67, 0.96) | 0.017* |
|  |  |  |  |  |
| Executive function |  |  |  |  |
| *Updating* | 0.91 (0.76, 1.09) | 0.319 | 0.90 (0.77, 1.05) | 0.172 |
| *Attentional-switching* | 1.03 (0.85, 1.25) | 0.759 | 1.05 (0.91, 1.22) | 0.488 |
| *Attentional-control* | 0.96 (0.84, 1.10) | 0.569 | 0.95 (0.82, 1.10) | 0.491 |
|  |  |  |  |  |
| Impulsivity | 1.21 (1.00, 1.50) | 0.047* | 1.19 (1.01, 1.42) | 0.042* |
|  |  |  |  |  |
| Sensation-seeking |  |  |  |  |
| *Arnett intensity subscale* | 1.17 (0.99, 1.38) | 0.073 | 1.17 (1.00, 1.37) | 0.048* |
| *Arnett novelty subscale* | 0.97 (0.83, 1.13) | 0.706 | 0.96 (0.83, 1.11) | 0.589 |
|  |  |  |  |  |
| Big-5 personality dimensions |  |  |  |  |
| *Extraversion (first quintile: lowest), n (%)* | 0.75 (0.48, 1.15) | 0.183 | 0.81 (0.54, 1.22) | 0.316 |
| *Extraversion (mid quintiles), n (%)* | - |  | - |  |
| *Extraversion (last quintile: highest), n (%)* | 1.23 (0.76, 1.99) | 0.409 | 1.19 (0.75, 1.88) | 0.460 |
| *Agreeableness* | 0.91 (0.74, 1.12) | 0.366 | 0.87 (0.72, 1.06) | 0.175 |
| *Conscientiousness* | 0.82 (0.69, 0.99) | 0.034* | 0.84 (0.71, 0.99) | 0.046* |
| *Emotional stability* | 0.99 (0.81, 1.20) | 0.895 | 0.92 (0.77, 1.10) | 0.354 |
| *Intellect/openness to experience* | 0.94 (0.78, 1.14) | 0.537 | 0.89 (0.74, 1.06) | 0.187 |
|  |  |  |  |  |
| Self-harm in friends and family |  |  |  |  |
| *Parent suicide attempt* | 1.97 (0.75, 5.19) | 0.172 | 1.65 (0.71, 3.84) | 0.247 |
| *Family self-harm (child-rated)* | 1.75 (1.20, 2.56) | 0.004** | 1.95 (1.39, 2.75) | <0.001** |
| *Friend self-harm (child-rated)* | 3.01 (2.06, 4.41) | <0.001** | 2.61 (1.85, 3.68) | <0.001** |
| Extent of exposure  *Friend/family only*  *Both* | 3.58 [2.29, 5.57]  5.18 [2.96, 9.06] | <0.001**  <0.001** | 3.21 [2.14, 4.82]  5.26 [3.17, 8.74] | <0.001**  <0.001** |
|  |  |  |  |  |
| Number of life events | 1.13 (0.98, 1.30) | 0.088 | 1.18 (1.04, 1.33) | 0.011* |
| Childhood sexual abuse | 1.84 (0.30, 11.1) | 0.509 | 1.30 (0.29, 5.71) | 0.732 |
| Cruelty to children in household | 1.67 (0.87, 3.19) | 0.122 | 1.71 (0.93, 3.11) | 0.082 |
| Being Bullied | 1.16 (0.81, 1.67) | 0.417 | 1.26 (0.91, 1.73) | 0.162 |
| Body dissatisfaction | 1.65 (1.16, 2.33) | 0.005** | 1.70 (1.24, 2.34) | 0.001** |
|  |  |  |  |  |
| **Psychiatric/mental health variables** |  |  |  |  |
| DAWBA diagnosis |  |  |  |  |
| *Depressive disorder* | 3.70 (1.49, 9.18) | 0.005** | 3.63 (1.67, 7.89) | 0.001** |
| *Anxiety disorder* | 1.75 (0.80, 3.84) | 0.161 | 2.20 (1.12, 4.30) | 0.022* |
| *Behavioural disorder (ODD/CD/ADHD)* | 2.85 (1.44, 5.64) | 0.003** | 2.90 (1.54, 5.44) | 0.001** |
|  |  |  |  |  |
| Depressive symptoms | 1.00 (0.88, 1.14) | 0.971 | 1.09 (0.97, 1.22) | 0.157 |
| Hopelessness | 1.39 (1.00, 1.92) | 0.047* | 1.47 (1.10, 1.98) | 0.010* |
|  |  |  |  |  |
| Substance use |  |  |  |  |
| *Alcohol (heavy drinking)* | 1.16 (0.75, 1.81) | 0.502 | 1.23 (0.82, 1.84) | 0.321 |
| *Cannabis (occasional)* | 1.24 (0.73, 2.12) | 0.433 | 1.15 (0.70, 1.89) | 0.578 |
| *Smoking (weekly)* | 2.34 (1.38, 3.98) | 0.002** | 2.54 (1.61, 4.02) | <0.001** |
| *Other illicit drug use (past year) ^a^* | 1.93 (1.20, 3.10) | 0.007** | 1.80 (1.18, 2.75) | 0.006** |

*^a^ Other illicit drug use does not include cannabis; Continuous risk factors are standardised (Z scores); * P <0.05; ** P <0.01*

**Appendix S1.** Missing data

As with all prospective studies, there was loss-to-follow-up with not all participants providing the necessary information for our analyses. We have compared responses to responders and non-responders to the age 16 year questionnaire and shown that data is not MCAR (missing completely at random). Those who responded to the questionnaire were more likely than those who did not to be female and white and to have lower parity (assessed during pregnancy), a mother with higher education (assessed during pregnancy), a higher household income, and a higher parental social class (assessed during pregnancy), and less likely to have experienced over-crowding (assessed during pregnancy) (see Supplementary Table 1). This can introduce selection bias and raises questions about the validity of the complete-case estimates.

To account for this possibility, our primary analyses were conducted on an imputed dataset based on those with complete outcome data (suicidal thoughts and attempts) at age 16 years (n=4772). Multivariable Imputation by Chained Equations (Royston and White, 2011) in Stata was used to create multiple copies of datasets in which missing values are replaced by imputed values, sampled from their predictive distribution. This method assumes data are Missing at Random (MAR), whereby any systematic differences between the missing and the observed values can be explained by differences in observed data (Sterne et al., 2009). One hundred imputed datasets were generated. All variables used in the analysis were included in the imputation models along with a number of additional auxiliary variables. These included variables found to be predictive of missingness (see supplementary table 1), indicators of socioeconomic adversity, personal characteristics, and maternal psychopathology (depression, substance use) as well as strong correlates of the risk factors, such as similar measures collected earlier or later in the study (for example psychiatric diagnosis at age 18 years, bullying at age 8 and 10 years, earlier measures of substance use and depression symptoms).

Under the missing at random assumption, multiple imputation should correct biases that may arise in the complete case analyses. This assumption is not testable and we therefore cannot say with certainty that the data are MAR. However, our imputation models included a wealth of auxiliary information, which increases the plausibility of the MAR assumption, including covariates known to be associated with non-response (Supplementary Table 1).

We compared the findings using imputed data with our complete case analysis and effect estimates were broadly consistent (Supplementary Table 4)

**References:**

ROYSTON, P. & WHITE, I. R. (2011). Multiple Imputation by Chained Equations (MICE): Implementation in Stata. *Journal of Statistical Software,* 45**,** 1-20.

STERNE, J. A., WHITE, I. R., CARLIN, J. B., SPRATT, M., ROYSTON, P., KENWARD, M. G., WOOD, A. M. & CARPENTER, J. R. (2009). Multiple imputation for missing data in epidemiological and clinical research: Potential and pitfalls. *British Medical Journal.,* 338.
